# Quercetin’s Effects on Glutamate Cytotoxicity

**DOI:** 10.3390/molecules27217620

**Published:** 2022-11-07

**Authors:** Kade Riche, Natalie R. Lenard

**Affiliations:** Department of Biology, School of Arts and Sciences, Franciscan Missionaries of Our Lady University, 5414 Brittany Drive, Baton Rouge, LA 70808, USA

**Keywords:** glutamate, excitotoxicity, reactive oxygen species, reactive nitrogen species

## Abstract

The potentially therapeutic effects of the naturally abundant plant flavonoid quercetin have been extensively studied. An extensive body of literature suggests that quercetin’s powerful antioxidant effects may relate to its ability to treat disease. Glutamate excitotoxicity occurs when a neuron is overstimulated by the neurotransmitter glutamate and causes dysregulation of intracellular calcium concentrations. Quercetin has been shown to be preventative against many forms of neuronal cell death resulting from glutamate excitotoxicity, such as oncosis, intrinsic apoptosis, mitochondrial permeability transition, ferroptosis, phagoptosis, lysosomal cell death, parthanatos, and death by reactive oxygen species (ROS)/reactive nitrogen species (RNS) generation. The clinical importance for the attenuation of glutamate excitotoxicity arises from the need to deter the continuous formation of tissue infarction caused by various neurological diseases, such as ischemic stroke, seizures, neurodegenerative diseases, and trauma. This review aims to summarize what is known concerning glutamate physiology and glutamate excitotoxic pathophysiology and provide further insight into quercetin’s potential to hinder neuronal death caused by cell death pathways activated by glutamate excitotoxicity. Quercetin’s bioavailability may limit its use clinically, however. Thus, future research into ways to increase its bioavailability are warranted.

## 1. Introduction

Much research at the intersection of the nutritional and health sciences has been conducted on the beneficial effects of flavonoids and polyphenols in preventing diseases. The most notable and abundant flavonoid that has been thoroughly studied in in vitro and in vivo research is quercetin, which has been shown to hold many beneficial properties, such as anti-inflammation, antioxidant, anti-cancer, and anti-microbial capabilities. Given that quercetin is a polyphenol, it is a powerful antioxidant and free radical scavenger. Quercetin has proven particularly beneficial against disease states relating to certain types of neuronal cell death [1]. As one example and the focus of this review, the neurotransmitter glutamate can cause excessive neuronal excitation, leading to oxidative stress, damage, and eventual cell death [2]. For a brief background, glutamate is responsible for most excitatory neurotransmission and synaptic plasticity, depolarizing neuronal cell membranes that transmit action potentials [3]. During certain disease states, high concentrations of glutamate can cause excessive neuronal depolarization and influx of calcium, which leads to oxidative stress conditions, mitochondrial destruction, and activation of pathways that lead to cell death [4]. The condition of cell death and toxicity caused by glutamate’s profuse activation is coined glutamate excitotoxicity.

This review will focus on the role glutamate plays in the nervous system, how glutamate excitotoxicity (GE) causes neuronal cell death through various mechanisms, and how quercetin acts against vital steps in those cell death pathways in the attenuation of the cytolytic effects of GE. This review will outline quercetin’s observed and hypothesized protective actions against the mechanisms that lead to neuron death during GE mentioned in the previous paragraph. The discussion of all cell death possibilities and how they are attenuated by quercetin is the center of this review to unify and organize a mechanism of action for quercetin against neuron death for current and future research. A better understanding of quercetin’s neuroprotective properties will aid and inspire clinical research against neurological diseases that result in GE.

## 2. Glutamate in the Central Nervous System

### 2.1. Role and Purpose

**Figure 1:**
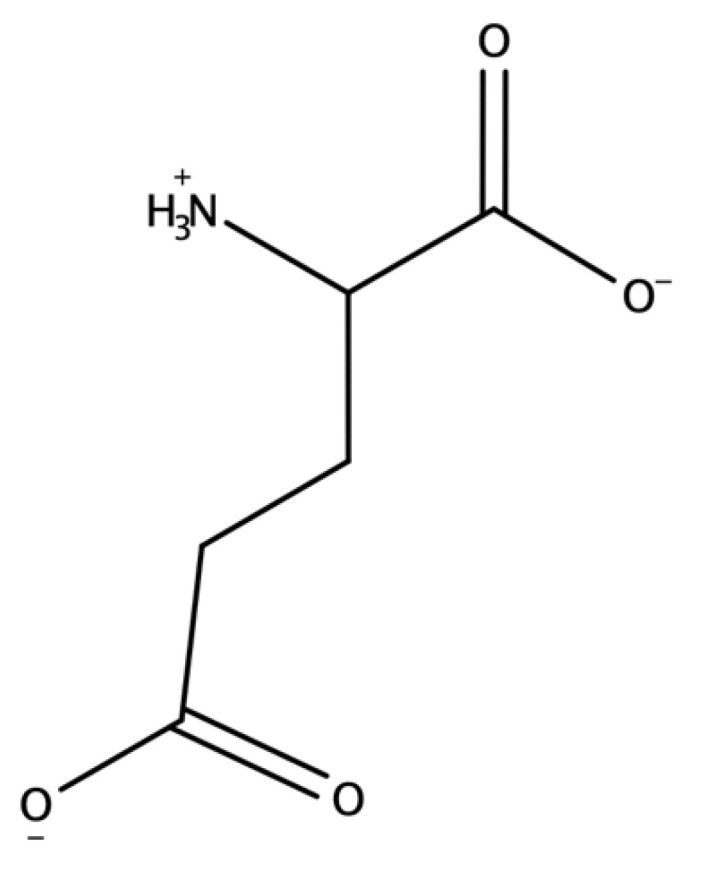
Structure of Glutamate.

Glutamic acid is a nonessential amino acid and is one of the brain’s amino acid neurotransmitters [4,5]. It exists in its conjugate base form, glutamate, at physiological pH. Like all amino acids, glutamate has a central carbon atom bonded to an amino group, carboxyl group, and R group. The R group that makes up glutamate is a CH_2_CH_2_COO^−^ (or CH_2_CH_2_COOH for glutamic acid), as seen in Figure 1. Glutamate’s functional groups, amino group, carboxyl group, and R group are all ionized at physiological pH.

Glutamate is the central nervous system’s (CNS) main excitatory neurotransmitter [6]. Glutamate’s function as an excitatory neurotransmitter in the brain arises from its ability to initiate action potentials by depolarizing postsynaptic neurons [7,8]. Glutamate’s excitatory function allows for many roles in the brain, including constructing neural connections and networks, synaptic plasticity, learning, and memory [3,9]. Over half of the brain’s neurons release glutamate, given that nearly all excitatory neurons in the CNS are glutamatergic [10]. 

### 2.2. Metabolism

Because the blood–brain barrier is impermeable to glutamate, glutamate is synthesized and recycled in the neurons and neuroglia through a heavily regulated process called the glutamate-glutamine cycle [10,11,12]. Glutamine is released from glial cells for neurons to use as the precursor to glutamate. The glutamine is taken in by the neurons at the presynaptic terminals and then converted to glutamate by the enzyme glutaminase or phosphate-activated glutaminase (PAG) [12]. Once glutamate is synthesized, it is packaged into vesicles by vesicular glutamate transporters and is then released from the vesicles at the synaptic cleft during neurotransmission [11]. Once glutamate is released from the synaptic vesicles, it interacts with the receptors on the postsynaptic neuron, which causes a stimulatory response in the postsynaptic neuron. Glutamate is then transported out of the synaptic cleft and into the surrounding glial cells or presynaptic neurons. Glutamate is then converted back into glutamine by glutamine synthetase enzymes [11,12]. The glutamate-glutamine cycle ensures that glutamate concentrations are sufficient for neurotransmission and balanced to ensure the prevention of neuronal toxicity.

### 2.3. Receptors and Signaling

Once glutamate is released from the presynaptic neuron, glutamate reacts with the receptors on the postsynaptic neuron to initiate postsynaptic neurotransmission, thus an excitatory effect [10]. Glutamate acts on multiple receptor types of two classes: ionotropic and metabotropic glutamate receptors [10]. Ionotropic glutamate receptors are ligand-gated ion channels, whereas metabotropic glutamate receptors utilize signal transduction and cascade pathways to modulate the neurotransmission [10]. Ionotropic glutamate receptors are the focus of this review because of their direct contribution to GE. 

The three ionotropic glutamate receptors expressed in most CNS neurons are separated into two groups–NMDA and non-NMDA receptors–named after the receptor’s synthetic, specific agonist [13,14,15,16]. The group of non-NMDA receptors consists of AMPA receptors and kainate receptors [13]. NMDA, AMPA, and kainate receptors differ in the speed it takes for the ion channels to open–with AMPA being the fastest, NMDA being the slowest, and kainate in between [17,18]. Though these receptors significantly differ in speed and function, they are all structurally similar: Four protein subunits each with three transmembrane regions and an extracellular region [13]. NMDA glutamate receptors differ in physiological function from the other glutamate receptors–AMPA and kainite–which makes them suitable targets for pharmaceutical therapies. Although AMPA and kainite receptors are permeable to Na^+^ and K^+^ to generate excitatory responses, NMDA is also permeable for Ca^2+^ ions that activate intracellular signaling cascades, making it the receptor shown to play a role in neuron plasticity [19]. NMDA receptors allow the passage of Na^+^ and Ca^2+^ ions inside the neuron and K^+^ outside the neuron, but glutamate binding is not the only initiating factor [13]. Unlike AMPA and kainate, multiple factors are required for the NMDA receptor ion channel to open aside from glutamate binding. At rest, the NMDA receptor ion channels in are blocked by magnesium (Mg^2+^) ions [10]. The Mg^2+^ is removed from the ion channel once the neuron’s cell membrane is depolarized [10,11]. At rest, the negative charge inside the neuron attracts the Mg^2+^ ion into NMDA’s channel pore. Once the cell membrane becomes depolarized, the positive charge inside the neuron repels the Mg^2+^ and it leaves the channel [10]. NMDA glutamate receptors can only conduct their ion passage when glutamate binds to NMDA while the cell membrane is depolarized to ensure NMDA’s channel opens at the same time Mg^2+^ is released, which is why NMDA glutamate receptors are known as coincidence detectors [13]. The last requirement for activating NMDA glutamate receptors is the binding of a co-agonist–either glycine or D-serine–on the GluN1 subunit, whereas glutamate binds to NMDA’s GluN2 subunit [13].

The two non-NMDA, AMPA and kainate, are responsible for most of the excitatory neurotransmission in the CNS. Given that non-NMDA glutamate receptors do not rely on membrane depolarization and are not blocked by Mg^2+^ like NMDA receptors, the movement of sodium and potassium ions through the glutamate-activated channels is natural. AMPA receptors are responsible for the most excitatory, postsynaptic action potentials in synapses [13]. AMPA receptors undergo structural changes after the binding of glutamate that causes the opening of its ion channel to allow the passage of Na^+^ and K^+^ in and out of the neuron [10]. The direct effects of glutamate on non-NMDA receptors make their neurotransmission faster than that of NMDA receptors.

Non-NMDA receptors do not require the binding of co-agonists and membrane depolarization to initiate an excitatory response on the postsynaptic membrane by glutamate. Evidence shows that some AMPA glutamate receptors are Ca^2+^ permeable if they do not contain specific subunits [13,20]. Postsynaptic membranes have different ratios of both NMDA and AMPA receptors. Suppose a single postsynaptic membrane with both NMDA and AMPA receptors is activated by several presynaptic, high-frequency action potential bursts. In that case, the postsynaptic currents caused by AMPA receptor activation could adequately keep the postsynaptic membrane depolarized enough to allow the de-blockage of NMDA receptors from magnesium ions [13].

In contrast, a single action potential and glutamate release brought on by the presynaptic neuron will only cause activation of AMPA receptors when the postsynaptic membrane is at rest [13]. When postsynaptic cells express only NMDA glutamate receptors, they are known as silent synapses because the postsynaptic cell can only contribute to neurotransmission or activity when the cell is in a depolarized state. Simultaneously, glutamate is released. Specific neuronal activity that causes silent synapses with NMDA glutamate receptors to contribute to neurotransmission has been proposed for synaptic plasticity [13]. Kainate receptors mediate excitatory postsynaptic potentials that are smaller and slower than those of AMPA receptors [13].

## 3. GE

### 3.1. Background

GE is described as the phenomenon when high concentrations of glutamate accumulate in the synaptic cleft, and excessive triggering of glutamate receptors causes the accumulation of Ca^2+^ inside the neuron and the activation of pathways that result in cell death. Neuronal death from prolonged postsynaptic excitatory neurotransmission from glutamate accumulation was discovered in 1957 when Lucas and Newhouse observed neuron death in mouse retinas after being fed sodium glutamate [21]. GE can occur secondary to neurological events, like trauma, ischemia, stroke, hypoglycemia, and epilepsy during neurodegenerative diseases (Alzheimer’s, Huntington’s, and Parkinson’s) [22,23,24,25,26,27].

In the case of certain disease states like ischemic stroke, the affected tissues in the brain differ based on the type of cell death. In the example of ischemic stroke, the direct region of the brain that is vascularly occluded will cause neuronal death by necrotic type death based on the direct blockage of O_2_, glucose, amino acids, and metabolic substrates needed for cellular functioning. While specific infarcted areas of the brain die, the surrounding tissues and neurons will be affected by the subsequent decrease in neurological functioning. They will overcompensate by releasing more neurotransmitters, especially glutamate [28]. In other disease states, many cellular mechanisms become defective and lead to overexcitement of glutamate, such as decreased glutamate clearance from microglia, fluxes in glutamate metabolism, and increased glutamate receptor sensitivity [2,27].

### 3.2. Importance of Calcium in GE

**Figure 2:**
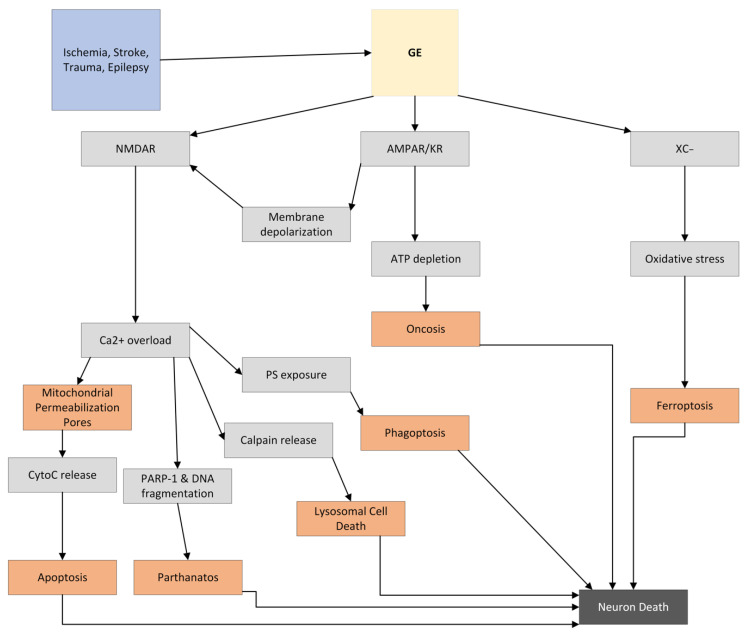
Diagram of the main effects of glutamate toxicity leading to the different forms of neuronal death. Abbreviations: NMDAR, NMDA receptor; AMPAR/KR, AMPA receptor/kainate receptor; x_c_^−^, glutamate/cysteine antiporter; Ca^2+^, calcium; CytoC release, cytochrome c release; PARP-1, Poly [ADP-ribose] polymerase 1.

GE and resulting neuronal death occur through many mechanisms. As discussed in 2.4, Ca^2+^ flows into neurons through NMDA receptor ion channels under the correct conditions. There are numerous ways that high Ca^2+^ concentrations result in neuronal cell death, with the most detrimental being Ca^2+^’s effect on the mitochondrial membrane potential (MMP). Overwhelming intracellular concentrations of Ca^2+^ can also cause the production of free radicals and upregulate cytotoxic transcription factors. In addition, Ca^2+^-dependent enzymes, such as phospholipase A_2_, cyclooxygenase-2, lipoxygenases, protein kinase C, calpain, xanthine oxidase, proteases, endonucleases, phospholipases, and lipases are activated [2,29,30]. Aside from producing free radicals, these enzymes can break away the cell’s membranes, leave it vulnerable to deadly chemicals and compounds, and break down necessary macromolecules needed for cellular functions [28]. Ca^2+^ overload is toxic to neurons not only by the direct role they play in activating destructive enzymes, but also by effects on mitochondria. The excess Ca^2+^ and its effects on the neuronal mitochondria are just the start of many routes that affect the neuron leading to its death. During GE, many cytolytic pathways are activated with or from the overload in calcium, including the intrinsic apoptosis pathway, mitochondrial permeability transition, lysosomal cell death, necrotic type cell deaths (oncosis, parthanatos, phagoptosis), ferroptosis, and reactive oxygen species (ROS)/reactive nitrogen species (RNS)-induced death, which are all outlined in Figure 2.

## 4. Quercetin

### 4.1. Background

Quercetin (2-(3,4-dihydroxy phenyl)-3,5,7-trihydroxychromen-4-one) is an abundant phytochemical and a flavonol abundantly found in such plant parts as fruits, vegetables, teas, seeds, nuts, flowers, and leaves [31,32]. Quercetin’s higher concentrations can be found in onions, apples, broccoli, tea, and red wine [33]. Table 1 outlines food sources of quercetin and its normalized amount based on the USDA (United States Department of Agriculture) Database for the Flavonoid Content of Selected Foods [34]. Quercetin has various biological and chemical properties that have been widely studied in biochemical, microbial, pathological, and clinical research. Flavonoids, especially quercetin, have anti-inflammatory, antioxidant, anti-carcinogenic, anti-viral, and many more properties that have been observed to promote cardiovascular, neurological, immunological, and systemic health. Studies have focused on quercetin’s ability to act as a strong antioxidant and anti-inflammatory, making it prevalent in numerous in vitro research on cardiovascular diseases, cancer, and neurological diseases. In vitro, quercetin has shown potent antioxidant capacities stemming from its polyphenol structure, but few in vivo applications have succeeded because of quercetin’s low bioavailability [35].

### 4.2. Chemical Properties

**Figure 3:**
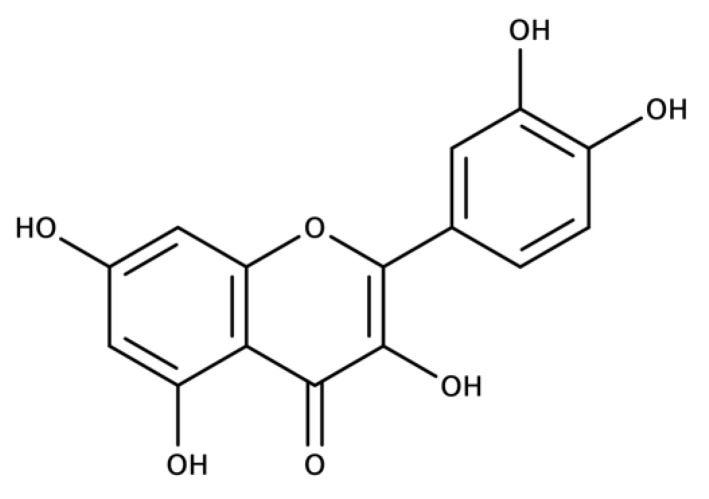
Structure of Quercetin.

Quercetin can also be referred to as quercetin aglycone because the pure structure is C_15_H_10_O_7_, as seen in Figure 3, and is a natural aglycone [36]. Quercetin is a pentahydroxyflavone with five hydroxyl groups at the 3, 3′, 4′, 5, and 7 positions and is categorized as a flavonol, which is specific to the class of flavonoid polyphenols. Quercetin is naturally lipophilic and soluble in alcohol but insoluble in water. Many quercetin conjugates can be found in nature and in the body; the most relevant and abundant are the quercetin glycoside, sulfated quercetin, and methylated quercetin, with quercetin glycoside being the most abundant in nature [35,37]. A glycosyl group commonly replaces the typical 3-hydroxyl group in quercetin glycoside, and effect that increases quercetin’s water solubility and overall absorption [36].

### 4.3. Bioavailability and Metabolism

Bioavailability refers to the chemical’s rate of absorption and capacity for utilization at a physiological level inside the body. Quercetin has been shown to be unstable and poorly absorbed in the gastrointestinal (GI) tract. The small amount of quercetin conjugates taken in by the GI’s epithelium is quickly metabolized by the liver, giving quercetin a short biological half-life of 1–2 h [38]. Thus, only a small amount of quercetin that is not metabolized is available to the body. Given that quercetin glycosides are naturally occurring in nature, they are the most ingested forms of quercetin. Quercetin glycosides are converted into quercetin aglycones by beta-glucosidases in the GI tract [39]. Once in the epithelium, the quercetin aglycones are converted into multiple derivates; the most common are quercetin conjugates that are either sulfated, methylated, or glucuronidated [39]. Quercetin glycosides are found in higher circulating concentrations, hypothesized to be due to sodium-dependent glucose transporters in the GI epithelium [38]. Although quercetin and its metabolites have been studied and calculated in circulation, it has been observed that quercetin’s bioavailability is too low for quercetin’s health benefits to take effect in the brain, especially given that the blood–brain barrier is impermeable to quercetin [40]. Therefore, overcoming quercetin’s low bioavailability and half-life will be critical in the application of its beneficial in vivo effects to attenuating GE. Future basic research should aim to determine and evaluate methods that increase quercetin’s potential clinical efficacy.

### 4.4. Quercetin’s Protective Mechanisms against Glutamate Excitotoxic Cell Death

Quercetin has been shown to combat neurotoxicity and neurodegenerative diseases by protecting and promoting cellular defenses against oxidative stress, mitochondrial dysfunction, neuroinflammation, and apoptosis [37]. During GE, neurotoxicity primarily occurs secondary to a rise in ROS and apoptotic pathway activation. Here, we will focus on the mechanisms of cell death resulting from GE and discuss quercetin’s observed and proposed neuroprotective effects on the cell death mechanisms, which are all illustrated and summarized in Table 2.

#### 4.4.1. Apoptosis

The catastrophic stimuli that initiate cytolytic cascades collectively known as apoptosis were initially observed as a singular event that leads to changes in the cell’s chromatin, organelle membranes, size, and production of vesicular bodies [41]. We now know apoptosis to be separated into either extrinsic or intrinsic forms depending on the factor causing the programmed death. Intrinsic apoptosis, also known as mitochondrial-initiated apoptosis, is the main form of apoptotic death induced by GE. Mitochondria play vital roles in functioning cells, such as maintaining intracellular Ca^2+^ concentrations and producing endogenous ROS. The intrinsic apoptosis pathway operates on B-cell lymphoma (Bcl-2) protein activity inside cells. The Bcl-2 proteins are arranged in different classes and are described as either pro-apoptotic or anti-apoptotic. The three anti-apoptotic proteins are Bcl-2, Bcl-x, and Bcl-w; In contrast, the pro-apoptotic proteins in the family consist of Bax, Bak, Bim, Bid, Bad, Noxa, and p-53 upregulated modulators of the apoptosis [42].

Inside neurons, intrinsic apoptosis pathways depend on the expression and activation of Bax proteins specifically [42,43]. Once neurons receive death signals–like increased Ca^2+^ levels, p53 activation, and FADD protein activation–to initiate the death cascade, BH3-only proteins can activate Bax and Bak proteins that stimulate Ca^2+^ mitochondrial entry via mitochondria calcium uniporters [44,45,46]. The Bax proteins, inactive monomers, undergo mitochondrial translocation and form active Bax oligomers on the outer mitochondrial membrane, which prompts mitochondrial outer membrane permeabilization because of Ca^2+^ overload and cytochrome c release [47,48,49]. The highly expressed neuronal cysteine protease, calpain, is activated in the neuron during Ca^2+^ overload. Calpain plays a role in cleaving the Bax and Bid proteins in the mitochondria which contributes to the release of cytochrome c and apoptosis-inducing factors from the mitochondria [50,51]. Though the exact mechanism is unknown, it has been observed that pores form on the outer mitochondrial membrane after Bax oligomerization and unknowingly disrupt complex II in the electron transport chain (ETC), which contributes to the depletion of ATP in surrounding tissues [43]. Once the ETC is disrupted in the mitochondria, more ROS are synthesized, which causes the downstream activation of OMA-1 proteins [43]. The OMA-1 proteins cleave and activate the mitochondria’s OPA-1L into OPA-1S [43]. The now active OPA-1S alters the mitochondria’s cristae to prompt the release of cytochrome c from the created pores [52].

Although the conditions under GE have implemented several cascades and predispositions that are toxic to the neuron’s survival, the primary motivators for cell death are the damaged mitochondria once they are met with an overwhelming concentration of Ca^2+^. Therefore, the ultimate rise in Ca^2+^ concentrations during GE leads to irreversible damage and the death of neurons and glial cells originates at the point of mitochondrial dysfunction. Once mitochondrial membrane permeabilization occurs and the pores in the mitochondrial membrane start to form, the release of cytochrome c and apoptotic protease activating factor 1 (Apaf-1) are prominent in the activation of the intrinsic apoptosis mechanism [53,54,55]. Cytochrome c and Apaf-1 are joined in an ATP-dependent manner and form what is known as an apoptosome. The apoptosome activates pro-caspase 9, becoming caspase 9, and further activates pro-caspase 3, becoming caspase 3 [53,54,55]. The active caspase 3 cleaves cellular proteins and substrates vital to cellular life, like microtubules, phospholipid membranes, DNA fragmentation, etc., causing cell death [53,54,55].

**Figure 4:**
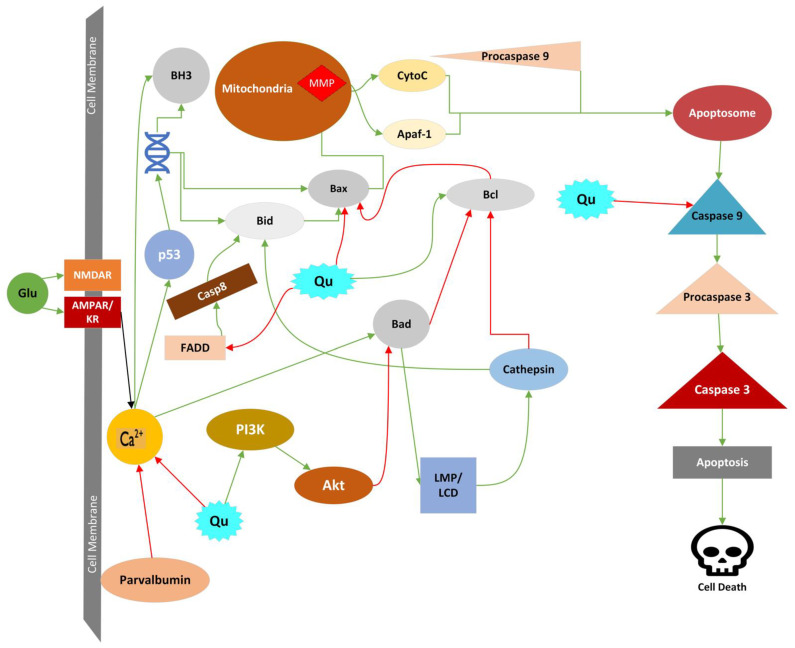
Diagram of quercetin’s neuroprotective properties against calcium-induced apoptotic mechanisms and LMP that are activated during GE. Green arrows indicate activation, and red arrows indicate inhibition. Abbreviations: Glu, glutamate; NMDAR, NMDA receptor; AMPAR, AMPA receptor; KR, kainate receptor; Ca^2+^, calcium; Qu, quercetin; CytoC, cytochrome c; Apaf-1, apoptotic protease activating factor 1; BH3, BH3-only proteins, MMP, mitochondrial membrane permeabilization; LMP/LCD, lysosomal membrane permeabilization/lysosomal cell death.

Quercetin possesses significant anti-apoptotic properties in neurons suffering from neurodegenerative diseases, ischemic stroke, or GE. Many of the cellular conditions during GE activate apoptotic mechanisms by various methods, most of which can be counteracted by quercetin, as seen in Figure 4, showing quercetin to be a beneficial therapy against GE. Firstly, quercetin has been shown to upregulate PI3K/AKT pathways in ischemic rat cells, leading to decreased expressed, apoptotic caspase 3 enzymes [40]. Quercetin can direct inhibition and downregulation of caspase 3 activation, which protects cells from intrinsic apoptosis mechanisms [56,57,58,59]. Aside from caspase 3 inhibition, quercetin has been shown to prevent intrinsic apoptosis by regulating Bcl-2 and Bax proteins [56,60]. Quercetin can lower Bax directly and inhibit FADD activity, inhibiting the activation of caspase 8 and caspase 8’s activation of Bid [57]. Lastly, quercetin has been observed to decrease calpain activity which halted the breakdown of neuronal macromolecules during apoptosis [61].

#### 4.4.2. Mitochondrial Permeability Transition

A mitochondrial permeability transition is a distinct form of cell death that occurs when the mitochondria’s inner membrane becomes permeable, mainly as a secondary response to high Ca^2+^ concentrations [2]. During the mitochondrial permeability transition process, mitochondrial permeability transition pores cause openings in the inner mitochondrial membrane, making the mitochondrion’s membrane freely permeable to various molecules and metals [43]. When the mitochondrial permeability transition pores open, and the inner mitochondrial membrane becomes depolarized, the mitochondria fall victim to ATP depletion, inhibition of oxidative phosphorylation, reversal of ATP synthase, swelling of the mitochondrial matrix, and release of cytochrome c, ultimately leading to cell death [42]. After mitochondrial membrane permeabilization, the available mitochondrial and cellular ATP concentrations diminish significantly. Depending on the cell’s initial condition, the ATP depletion after mitochondrial membrane permeabilization can cause necrosis if the cell is predisposed to ATP-reducing factors, such as ischemia and sometimes GE [42]. During ischemia, GE, and other pathological states, the neuron’s available ATP concentrations can be depleted before the mitochondrial permeability transition, which causes necrotic cell death [62]. Given that apoptosis requires ATP to build apoptosomes, neurons without adequate amounts of ATP cannot undergo apoptosis after cytochrome c release and caspase activation [43].

**Figure 5:**
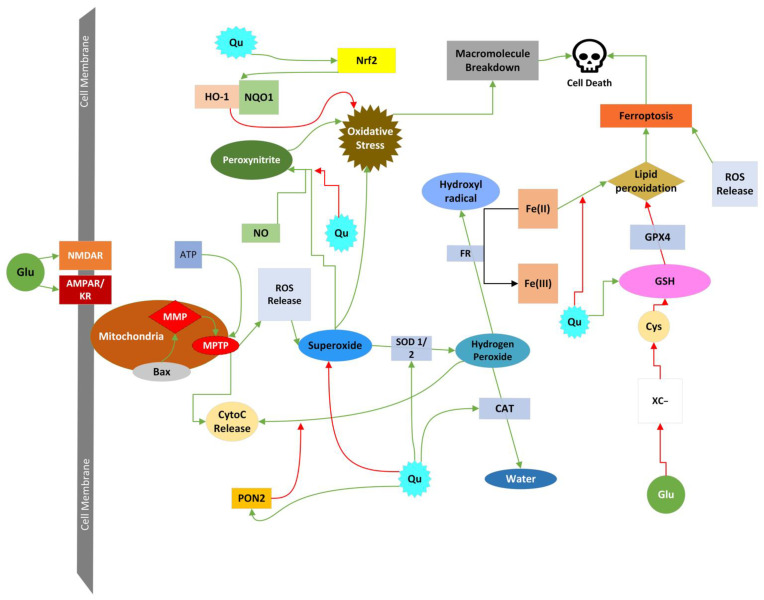
Diagram of quercetin’s neuroprotective properties against oxidative damage from ROS/RNS, ferroptosis, and mitochondrial membrane permeabilization that occurs during GE. Green arrows indicate activation, and red arrows indicate inhibition. Abbreviations: Glu, glutamate; NMDAR, NMDA receptor; AMPAR, AMPA receptor; KR, kainate receptor; Qu, quercetin; CytoC, cytochrome c; MMP, mitochondrial membrane permeabilization; MPTP, mitochondrial permeability transition pores; ROS, reactive oxygen species; NO, nitric oxide; SOD1/2, superoxide dismutase 1 or 2; CAT, catalase; HO-1, heme oxidase 1; NQO1, NADPH dehydrogenase quinone-1; Nrf2, nuclear factor erythroid 2-related factor 2; FR, Fenton Reaction; Xc^−^; cystine/glutamate exchanger; Cys, cysteine; GSH, glutathione; GPX4, glutathione peroxidase 4; ATP, adenosine triphosphate.

As seen in Figure 5, quercetin can protect cells from mitochondrial injury through reducing the damage Ca^2+^ overload has on the mitochondrial membrane. Quercetin protects cells from Na^+^/K^+^-ATPase pump deficiency and membrane depolarization resulting from upregulation of parvalbumin proteins. Parvalbumin is a protein that binds and neutralizes Ca^2+^ when it enters neurons to ensure that overload does not occur during neurotransmission. Quercetin has been shown to increase cellular parvalbumin concentrations during neuronal stress, suggesting that it has promising neuroprotective effects in counteracting the Ca^2+^ overload [63]. Along with reducing cytochrome c concentrations, quercetin has been shown to reduce the concentration of Ca^2+^ in hippocampal neurons after ischemic injury [56]. There is also evidence showing that quercetin protects against mitochondrial membrane permeability from the Ca^2+^ injury [64].

#### 4.4.3. Lysosomal Cell Death (LCD)

LCD or autolysis is cell death resulting from lysosomal membrane permeabilization (LMP). Lysosomes are the digestive organelles for cells that function at low pH to break down macromolecules, dysfunctional organelles, and extracellular particles by using proteases, hydrolases, and other degradative enzymes, including cathepsins [65,66]. Lysosomal cell death occurs when the lysosome’s membrane becomes permeable to leakage and releases the degradative enzymes into the cytosol, breaking essential macromolecules and secondary effects leading to LCD. During ischemia or GE, the intracellular Ca^2+^ overload contributes highly to LMP. However, many factors contribute to LMP during events that cause cellular stress. Other provokers of LMP include the activation of caspases, Bid and Bax-induced pore formation, phospholipase A_2_, lysosomal p53, oxidative stress, and activating calpains [67,68,69,70]. All these factors generate LMP, which allows the release of terminal substances from the lysosome that include enzymes like the protease cathepsins and DNase II, free protons that cause acidification of the cell, and ROS [43]. The degradation of the membrane and the release of lysosomal contents add to the oxidative stress and cellular damage occurring during the ischemia and GE [44,62]. Quercetin’s inhibition of Bax, activation of Bcl-2, and decrease in Ca^2+^ concentrations that lead to the reduction of ROS and RNS provide neuronal protection and the potential to halt cell death during LMP, as shown in Figure 4 [40].

#### 4.4.4. Oncosis

Derived from the Greek word for swelling, oncosis has been referred to in medical sciences as the type of cell death caused by intracellular swelling [71,72]. The process of oncosis has been further studied and observed in cell death induced after ischemic conditions and resulting from cellular ATP depletion and swelling [43,71]. Oncosis is a central mode of ischemic cell death, but, as we know, ischemic conditions can also cause mitochondrial dysfunction [42,43]. The determination of cell death depends on the intracellular ATP concentration because ATP synthesis depends on the mitochondrion’s membrane stability and the process of apoptosis requires ATP for building the apoptosome [42]. When neurons are deprived of ATP, they cannot maintain the electrochemical gradients necessary for proper neurotransmission and biochemical metabolism.

The neuron’s ion pumps (specifically Na^+^/K^+^ ATPases) cannot function without ATP, leading to an influx of Na^+^ and Ca^2+^ and K^+^ efflux, which causes unstimulated neuronal depolarization [28]. Once the neurons depolarize sufficiently, the L-type voltage-gated Ca^2+^ channels (VGCCs) are opened, leading to increased intracellular concentrations of Ca^2+^ ions [73]. Once glutamate binds to non-NMDA receptors, excitatory postsynaptic potentials are made that then open VGCCs to allow Ca^2+^ into the cell [53,54,55]. During this time, glutamate molecules can activate NMDA receptors because the neuron is depolarized from the malfunction in the Na^+^/K^+^ ATPase ion pumps. The elevated concentrations of Ca^2+^ inside the neurons prompt the release of glutamate extracellularly, and once glutamate binds to its NMDA receptors, more Ca^2+^ influx occurs. The subsequent rise in calcium concentrations can further cause mitochondrial permeability transition, activation of proteases and phospholipids, and other downstream signaling pathways that lead to the death of the neuron.

Although the areas surrounding the “zone of infarction” are not occluded from O_2_ and glucose, activating the intrinsic apoptosis pathway by GE is possible. During GE, excessive glutamate activity on non-NMDA receptors causes an increase in intracellular Na^+^ ions, which depletes the neuron’s available ATP when the Na^+^/K^+^-ATPase pump activity increases to compensate for the high Na^+^ concentration. The cytosolic ATP depletion, Na^+^ accumulation, intracellular swelling, Ca^2+^ pump failure, Ca^2+^ accumulation, and downfield activation of intrinsic apoptosis pathways and cytolytic enzymes result from glutamate activity on neuron’s postsynaptic receptors the oncotic type cell death [62].

#### 4.4.5. Parthanatos

**Figure 6:**
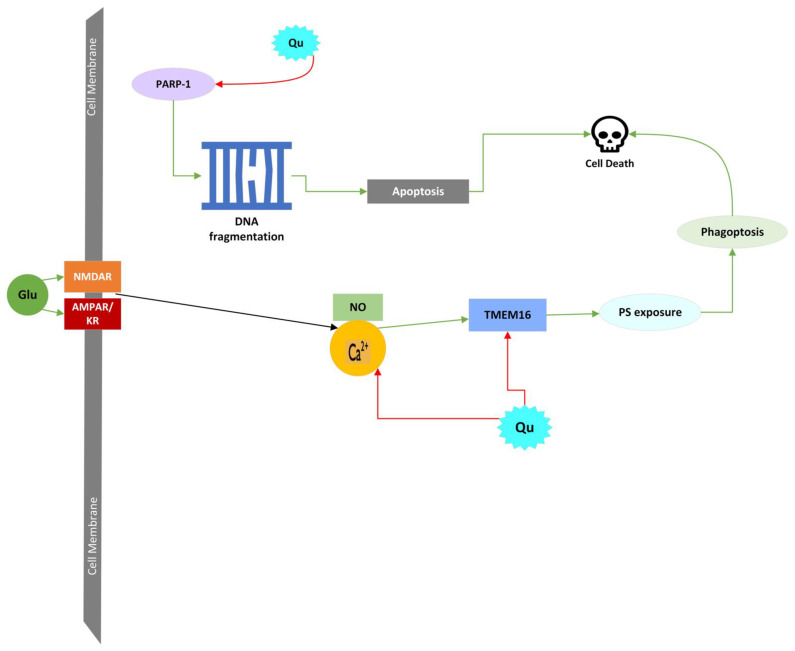
Diagram of quercetin’s neuroprotective properties against parthanatos and phagoptosis, which are activated during GE. Green arrows indicate activation, and red arrows indicate inhibition. Abbreviations: Glu, glutamate; NMDAR, NMDA receptor; AMPAR, AMPA receptor; KR, kainate receptor; Ca^2+^, calcium; Qu, quercetin; NO, nitric oxide; PS, phosphatidylserine; PARP-1, Poly [ADP-ribose] polymerase 1.

Parthanatos is another regulated form of necrotic cell death that is specific to the activity of poly(ADP-ribose) polymerase-1(PARP-1) inside the neuron [43,74]. The activation of PARP-1 is initiated by damage and stress to cellular DNA. Once PARP-1 is activated, it releases poly-ADP-ribose and depletes NAD^+^ in the nucleus [43]. Poly-ADP-ribose can release apoptosis-inducing factors (AIFs) and AIF: Cyclophilin A complexes from the mitochondria through inhibiting hexokinase enzymes or promoting the release from mitochondria, which ultimately ends in the fragmentation of nuclear DNA [75,76]. During GE, peroxynitrite concentrations are increased from nitric oxide and superoxide [77]. The peroxynitrite causes damage to the cell’s DNA, which initiates the parthanatos cell death pathway [43]. The direct effects quercetin can circumvent this type of necrotic cell death through decreasing the levels of PARP-1 inside the cell, which can desensitize the cell to the mechanisms of parthanatos necrotic cell death. Quercetin-induced increases in PI3K can downregulate PARP-1 and protect the neuron from its activation [78,79]. Figure 6 shows how quercetin can downregulate parthanatos during GE.

#### 4.4.6. Phagoptosis

Phagocytosis is a cell death outside the mechanisms of necrosis and apoptosis because it occurs when a living cell is phagocytosed by another cell (phagocyte) [43]. The process of phagocytosis occurs in three steps: recognition, engulfment, and digestion. The phagocytes can recognize the target cells by multiple cellular signals. The phagocytes express phagocytic receptors (like vitronectin receptors and Mertk) that bind to opsonins (like MFG-E8 and Gas6) on the target cells attached to well-known “eat me signals” like phosphatidylserine. Phosphatidylserine in the membranes of target cells can be exposed irreversibly or reversibly, leading cells to be phagocytosed. It is well known that the participation of caspase proteins results in the programmed death of the cell; therefore, when phosphatidylserine is exposed irreversibly, it is done so as an outcome of the caspase cleavage-mediated activation of a phosphatidylserine scramblase Xkr8 or by the caspase cleavage-mediated inhibition of the phosphatidylserine translocase, ATP11C [80,81]. When phosphatidylserine is exposed on the cell membrane, it is done by activating the TMEM16F scramblase because of the deficit in available ATP needed for the activity of translocases [82,83]. It has been shown that glutamate can cause the reversible exposure of phosphatidylserine on neurons, which can play a role in neuron death during conditions of excessive glutamate exposure (e.g., as in GE). Microglia can also reverse phosphatidylserine exposure by releasing ROS and RNS [84]. When phosphatidylserine is exposed, the Gas6 opsonins bind with the Mertk receptors, or the MFG-E8 opsonins bind with the vitronectin receptors, to initiate phagoptosis [85,86]. Depleting ATP inside a neuron accompanied by GE and ischemic injury prompts phosphatidylserine exposure. Given that phagoptosis relies on a phagocyte engulfing a target cell, for neuronal death to occur microglial cells must be present in the neuronal tissue during GE [84]. The reduction in ROS and Ca^2+^ concentrations induced by quercetin decreases the expression of TMEM16 to stop phosphatidylserine exposure, as shown in Figure 6. Effective ROS scavenging and downstream inhibition of the “eat me” signals aid in the halt of cell death by phagocytosis in the presence of microglia.

#### 4.4.7. Ferroptosis

Ferroptosis is a form of programmed cell death dependent on iron-induced lipid peroxidation of polyunsaturated fatty acids that results in necrotic cell death. Excessive accumulation of Fe^2+^ can cause lipid peroxidation, which can be toxic to neuronal membrane phospholipids. Lipid peroxidation can cause the production of ROS and free radicals that can break down the polysaturated fatty acids within cells, including the fatty acids that make up phospholipid membranes, and lead to poor membrane integrity and, ultimately, cell death [43]. Within neurons, lipid peroxidation is protected by glutathione peroxidase 4 (GPX4) enzymes. The protecting ability of glutathione peroxidase 4 depends on the availability of glutathione in the cell [87]. The glutathione inside the cell is acquired through the amount of cystine and cysteine. Cystine is broken down into cysteine, which is then turned into glutathione for glutathione peroxidase 4 [87]. The x_c_^−^, also known as the cystine/glutamate exchanger, is an antiporter that transports glutamate outside the cell and transports cystine inside the cell [85]. During ischemic conditions and GE, increased glutamate concentrations in the extracellular region of the neuron inhibit the x_c_^−^ antiporter activity [88]. Once the x_c_^−^ system’s activity is inhibited by glutamate, the neuron has decreased cystine availability, reducing the neuron’s available glutathione concentrations [89]. The depleted amounts of glutathione leave the neuron vulnerable to low glutathione peroxidase 4 availability and, thus, cell death induced by the lipid peroxidation [88,89]. As summarized in Figure 5, quercetin proves protective against iron-induced cell death, which can show beneficial effects during GE. Quercetin has been shown to prevent the activation of the neuron’s ferroptosis pathway by chelating reduced iron and increasing the expression of the glutathione peroxidase [90]. The subsequent increase of Nrf2 expression in neurons by quercetin can upregulate the expression of glutathione peroxidase 4 and SLCA11 enzymes that prevent the production of lipid radicals that are the product of iron toxicity [91].

#### 4.4.8. Cell Death Induced by ROS and RNS

Reactive molecules have been shown to play roles in many pathological and degenerative diseases. ROS are highly reactive molecules derived from oxygen (O_2_). The most notable intracellular ROS are superoxide, hydrogen peroxide (H_2_O_2_), hydroxyl radical, lipid radicals, and lipid peroxides. RNS are highly reactive molecules derived from nitric oxide (NO). The most notable RNS include NO, peroxynitrite, nitrogen dioxide, and S-nitrosothiols. ROS and RNS can freely react with any other molecule without using or managing cellular enzymes, causing oxidative stress within the cell and threatening the cell’s viability. Cells have defensive molecules and pathways to protect themselves from reactive molecules, including the glutathione pathway, ubiquinol, and antioxidant vitamins. When the number of reactive molecules exceeds the cell’s ability to neutralize the ROS or NOS, oxidative stress can break down macromolecules like DNA, proteins, lipids, and enzymes and initiate cellular death cascades. ROS can be generated through several routes in cellular metabolism, such as ETC complexes, lipoxygenases, NO synthases, xanthine oxidases, peroxisomal oxidases, NADPH oxidases, and more, depending on the cell type and environment [92]. NMDA receptors activate NADPH oxidase during excitotoxic neuronal injury, producing superoxide molecules [93,94,95]. Superoxide molecules can only damage neurons when they are in the presence of NO because superoxides and NO produce peroxynitrite, which is toxic to neurons [96,97,98]. Hydroxyl radicals serve as neurotoxic ROS through severely damaging macromolecules and can be made by superoxides and H_2_O_2_ in the presence of an iron [99,100]. As discussed in the process of phagoptosis, microglia can release ROS and RNS by NADPH oxidase enzymes to initiate phagocytosis of neurons during GE [101]. Neurons undergoing cell death by ferroptosis in response to GE create lipid radicals when the cystine/glutamate antiporter is inhibited, as mentioned in relation to ferroptosis [102].

Quercetin’s antioxidant effects in nervous tissue have been primarily studied in neurodegenerative diseases, ischemic injury, and neurovascular health research. During GE, the rise in concentrations of ROS, RNS, lipid peroxides, and free radicals contribute primarily to the damage of neurons and activation of cell-death pathways. Quercetin is a potent antioxidant based on its chemical structure and effects on antioxidant signaling pathways. Due to the catechol group in the B ring and the hydroxyl group at 3, quercetin can directly scavenge ROS like superoxide anions and hydroxyl radicals and scavenge RNSs like NO and peroxynitrite [103]. Moreover, quercetin strongly affects neuronal mechanisms that generate ROS and RNS and neuronal defense mechanisms that neutralize ROS and RNS. Glutathione metabolism is a convoluted pathway that allows cells to neutralize ROS and RNS arising from molecules produced during cell metabolism. Oxidative stress can occur if ROS, RNS, lipid peroxides, and free radicals rise above the cell’s glutathione concentrations, leading to cell damage and death if not counteracted. In vitro, quercetin protects neuronal cultures against H_2_O_2_ -induced oxidant stress through increasing glutathione levels [104]. Additional studies have observed a rise and maintenance in glutathione levels that protect against oxidative stress from ischemic conditions and glutamate-induced oxidative stress [1,105,106,107,108,109].

Quercetin has been shown to enhance levels of nuclear factor erythroid 2-related factor 2 (Nrf2), a protein that enhances neuronal production of enzymes that work as antioxidants [110,111,112,113]. Quercetin-induced increases in Nrf2 and control of its activation have been accompanied by the rise of heme oxidase-1 (HO-1), superoxide dismutase 1 and 2 (SOD 1–2), NADPH dehydrogenase quinone-1, glutathione, catalase, and glutathione peroxidase-1 (GPX-1) both in vitro and in vivo [113,114,115]. Studies measuring erythrocyte SOD activity in subjects with high dietary quercetin intakes have shown decrease systemic oxidative damage potential from increase SOD activity [116]. These enzymes play vital roles in neutralizing and protecting neurons from oxidative stress. In in vitro studies of microglia and neurons and in vivo studies of orally ingested quercetin, quercetin has been shown to increase paraoxonase 2 (PON2) and PON2 mRNA, which inhibits the conversion of superoxide to H_2_O_2_ within the mitochondria to prevent the release of cytochrome c [117,118,119,120]. Nanoencapsulated quercetin has been shown to reduce the expression of inducible nitric oxide synthase (iNOS) in ischemic rat hippocampus, an effect associated with downregulated synthesis of RNS and upregulated levels of glutathione, SOD1, and HO-1 [64]. Studies on reperfusion after ischemia in the brains of rats have shown decreased levels of malondialdehyde and myeloperoxidase, which downregulated the production of NO, ROS, and lipid peroxidation [40]. Figure 5 and Table 2 work to summarize quercetin’s protective effects against cell death caused by oxidative damage during GE.

With the rise in ROSs and RNSs that causes death to neurons during GE, quercetin’s protective abilities to scavenge free radical molecules and regulate enzymes that modulate the intracellular amount of ROS/RNSs can prove to be an effective therapy against cell death in the brain from GE.

## 5. Conclusions

Quercetin has been shown to protect, through various modalities, neurons undergoing GE. Quercetin reduces cellular calcium ion concentrations, a vital effect given that the primary toxicity of GE is due to calcium overload. Quercetin modulates many different proteins and enzymes responsible for several cell death pathways. In vivo imaging techniques on neuron death have hindered our ability to understand neuron death in its entirety, especially during pathological states. Effective imaging approaches towards neuron death in vivo during GE and how quercetin can attenuate processes during GE can lead to more comprehensive understandings of quercetin’s clinical significance. To properly utilize quercetin’s protective properties against GE in the clinical setting, future studies are needed to focus on efforts for increasing quercetin’s bioavailability, developing pharmacological deliverance methods for quercetin that maximizes its permeability through the blood–brain barrier, clarifying the effective dose of quercetin needed to protect against GE injury, and long-term in vivo studies that measure the effects quercetin has against GE injury in disease states. Certain studies suggest that future research should be directed towards observing quercetin’s in vivo effects towards synaptic plasticity and neurogenesis after ischemic stroke injury [40]. Studies on the pharmacological effects quercetin’s metabolites have could benefit our understanding of quercetin’s neuroprotective effects. 

## Figures and Tables

**Table 1:** Most Common Food Sources for Quercetin. Retrieved from the USDA (United States Department of Agriculture) Database for the Flavonoid Content of Selected Foods [34].

Source	Quercetin Amount (mg/100 mg)
Apples	4.01
Asparagus	14.0
Black tea	2.5 mg/100 mL
Blueberry	14.6
Broccoli	13.7
Cherry	17.4
Chili pepper	32.6
Chives	10.4
Cranberry	25.0
Dill	79.0
Fennel leaves	46.8
Kale	22.6
Lettuce	14.7
Onions	45.0
Oregano	42.0
Red wine	3.16 mg/100 mL
Spinach	27.2

**Table 2:** Summary of Quercetin’s Effect On Cell Death Pathways Caused by GE. Abbreviations: +, upregulate; −, downregulate.

Quercetin’s Effect (+/−)	Molecule/Protein Affected by Quercetin	Cell Death Pathway Affected
−	Intracellular Ca^2+^ increase	Apoptosis; LCD; Phagoptosis
+	Parvalbumin	Apoptosis
+	PI3K	Apoptosis; Parthanatos
−	NO	Oxidative Stress
−	PARP-1	Parthanatos
−	Cytochrome c	Apoptosis
+	Bcl	Apoptosis
−	FADD	Apoptosis
−	Bax	Apoptosis
−	Casp9	Apoptosis
+	Nrf-2	Oxidative Stress
−	Peroxynitrite	Oxidative Stress
+	PON2	Oxidative Stress
−	Superoxide	Oxidative Stress
+	Catalase	Oxidative Stress
+	SOD 1/2	Oxidative Stress
−	Fe(II)/Lipid Peroxide	Ferroptosis
+	Glutathione	Oxidative Stress; Ferroptosis
−	TMEM16	Phagoptosis

## Data Availability

Not applicable.
